# Urban physical disorder and alcohol consumption among adolescents in Brazilian capitals: a cross-sectional study

**DOI:** 10.1590/0102-311XEN032724

**Published:** 2025-04-28

**Authors:** Rayara Mozer Dias, Cláudia de Souza Lopes, Taísa Rodrigues Cortes, Kátia Vergetti Bloch, Washington Leite Junger

**Affiliations:** 1 Instituto de Medicina Social Hesio Cordeiro, Universidade do Estado do Rio de Janeiro, Rio de Janeiro, Brasil.; 2 Faculdade de Medicina, Universidade Federal do Rio de Janeiro, Rio de Janeiro, Brasil.

**Keywords:** Alcohol Consumption, Adolescent Health, Environmental Health, Urban Health, Consumo de Bebidas Alcoólicas, Saúde do Adolescente, Saúde Ambiental, Saúde da População Urbana, Consumo de Bebidas Alcohólicas, Salud del Adolescente, Salud Ambiental, Salud Urbana

## Abstract

This study aimed to estimate the association between urban physical disorder and alcohol consumption in Brazilian adolescents. The sample was composed of 2,384 adolescents, aged 12 to 17, resident in Brazilian capitals and participants in the *Study of Cardiovascular Risk in Adolescents* (ERICA), a school-based cross-sectional study undertaken in 2013 and 2014. The outcome variable was alcoholic beverage consumption characterized as having drunk an alcoholic beverage at least once in the previous 30 days. The exposure variable was urban physical disorder measured according to the urban features in the 2010 *Demographic Census*. The total effect of the indicators of exposure to urban physical disorder on the consumption of alcohol among adolescents was estimated using Poisson regression models with robust variance. Crude and adjusted prevalence ratios (PR) were calculated with 95% confidence intervals (95%CI) for each exposure, controlling for possible confounding variables. Adolescents living in areas with paved roads and manholes presented higher prevalence of alcohol consumption (adjusted PR = 1.24, 95%CI: 1.02; 1.50 and adjusted PR = 1.36, 95%CI: 1.01; 1.84, respectively). Conversely, the presence of wheelchair ramps and open sewers was associated with a lower prevalence of alcohol consumption (adjusted PR = 0.79, 95%CI: 0.62; 0.99 and adjusted PR = 0.80, 95%CI: 0.66; 0.97, respectively). These findings suggest that urban contextual factors can influence alcohol consumption among adolescents. Understanding these factors may aid in the development of public health policies that promote healthier urban environments.

## Abstract (pt)

Este estudo buscou estimar a associação entre desordem física urbana e consumo de álcool em adolescentes brasileiros. Sua amostra foi composta por 2.384 adolescentes com idades entre 12 e 17 anos que residiam nas capitais brasileiras e participaram do *Estudo de Riscos Cardiovasculares em Adolescentes* (ERICA), um estudo transversal de base escolar realizado entre 2013 e 2014. A variável de desfecho foi o consumo de bebida alcoólica, caracterizado pela ingestão de bebida alcoólica em pelo menos uma vez nos últimos 30 dias. A variável de exposição foi o distúrbio físico urbano, medido pelas características urbanas no *Censo Demográfico* de 2010. O efeito total dos indicadores de exposição à desordem física urbana sobre o consumo de álcool entre adolescentes foi estimado por modelos de regressão de Poisson com variância robusta. Foram calculadas razões de prevalência (RP) brutas e ajustadas por intervalos de 95% de confiança (IC95%) para cada exposição, controlando-se possíveis variáveis de confusão. Os adolescentes residentes em áreas com calçadas e em áreas com bueiros apresentaram maior prevalência de consumo de álcool (RP ajustada = 1,24, IC95%: 1,02; 1,50 e RP ajustada = 1,36, IC95%: 1,01; 1,84, respectivamente). Por outro lado, a presença de rampas para cadeiras de rodas e esgotos a céu aberto foi associada a uma menor prevalência de consumo de álcool (RP ajustada = 0,79, IC95%: 0,62; 0,99 e RP ajustada = 0,80, IC95%: 0,66; 0,97, respectivamente). Esses achados sugerem que fatores contextuais urbanos podem influenciar o consumo de álcool entre adolescentes, a compreensão dos quais pode auxiliar o desenvolvimento de políticas públicas de saúde para promover ambientes urbanos mais saudáveis.

## Abstract (es)

Este estudio tuvo como objetivo estimar la asociación entre el trastorno físico urbano y el consumo de alcohol en adolescentes brasileños. La muestra estuvo conformada por 2.384 adolescentes de entre 12 y 17 años que vivían en capitales brasileñas y que habían participado en el *Estudio de Riesgos Cardiovasculares en Adolescentes* (ERICA), un estudio transversal de base escolar realizado entre 2013 y 2014. La variable de resultado fue el consumo de alcohol, caracterizado por la ingesta de alcohol al menos una vez en los últimos 30 días. La variable de exposición fue el trastorno físico urbano medido por las características urbanas en el *Censo Demográfico del* 2010. El efecto total de los indicadores de exposición al trastorno físico urbano sobre el consumo de alcohol entre los adolescentes se estimó mediante los modelos de regresión de Poisson con varianza robusta. Se calculó la razón de prevalencia (RP) bruta y se ajustó mediante intervalos de 95% de confianza (IC95%) para cada exposición, con control de las posibles variables de confusión. Los adolescentes que viven en áreas con aceras y en áreas con alcantarillas tuvieron una mayor prevalencia de consumo de alcohol (RP ajustada = 1,24, IC95%: 1,02; 1,50 y RP ajustada = 1,36, IC95%: 1,01; 1,84, respectivamente). Por otro lado, la presencia de rampas para sillas de ruedas y alcantarillas abiertas se asoció con una menor prevalencia de consumo de alcohol (RP ajustada = 0,79, IC95%: 0,62; 0,99 y RP ajustada = 0,80, IC95%: 0,66; 0,97, respectivamente). Estos hallazgos indican que los factores contextuales urbanos pueden influir en el consumo de alcohol entre los adolescentes, lo que puede ayudar al desarrollo de políticas de salud pública para promover entornos urbanos más saludables.

## Introduction

In urban environments, urban physical disorder is known to be a health determinant, which is characterized by visual signs of negligence and uncontrolled deterioration, such as abandoned buildings, broken lampposts, and plots full of rubbish ^1^. The structure and dynamics of a city (standards of urban mobility, air quality, basic sanitation and housing, among other determinants) may significantly impact residents’ health ^2^.

Adolescent health may be particularly affected by frailty and dependence on parents/guardians, which can make them more vulnerable to their physical and social environment, particularly those of lower socioeconomic level ^3^. This life stage is marked by rapid changes in human development that require constant adaptations on the part of the individual and their context, which have a decisive impact on adolescent health, with both immediate and long-term implications for population health, given that adolescence is the onset of many risk-taking behaviors that can perpetuate throughout adulthood ^4^ ^,^ ^5^.

Adolescents are particularly prone to engage in health-risk behaviors. The literature presents risk behavior in adolescence as involvement in activities that can cause physical or mental harm, such as alcohol consumption ^6^ ^,^ ^7^. The 2019 *National Survey of School Health* (PeNSE) pointed to a worrisome scenario in Brazil, showing that 63.3% (95% confidence interval - 95%CI: 62.6; 64.0) of schoolchildren aged from 13 to 17 years had tried alcoholic beverages at some point in their lives, an increase compared to PeNSE 2015, which indicated 61.4% (95%CI: 59.3; 63.6) for this age group. There was also a change in the first contact with alcohol before the age of 13, increasing from 30.6% (95%CI: 28.7; 32.6) in 2015 to 34.6% (95%CI: 33.8; 35.3) in 2019 ^8^. 

Experimenting with alcohol in adolescence can have implications for an individual’s well-being and health throughout their lives, due to the greater risk of developing noncommunicable chronic diseases, such as high blood pressure, obesity and stroke ^9^ ^,^ ^10^. Studies have shown that the environment in which individuals circulate is among the main factors associated with alcohol consumption by adolescents, but evidence is still limited ^11^ ^,^ ^12^.

A review study demonstrated that, in Latin America, most recent publications on urban disorder and health outcomes were carried out on adults ^1^, highlighting the need for studies that investigate the association between urban environmental characteristics and alcohol consumption among younger populations. Therefore, this study aimed to estimate the association between urban physical disorder and alcohol consumption in Brazilian adolescents.

## Materials and Methods

### Study design, population, and time frame

This is a cross-sectional study with data from the *Study of Cardiovascular Risks in Adolescents* (ERICA), carried out in 2013 and 2014. ERICA is a multicenter, national study, which included 74,589 students from public and private schools. The authors describe the methodological procedures and further information about the study ^13^ ^,^ ^14^ ^,^ ^15^.

A subsample of adolescents residing in the state capitals of Rio de Janeiro (3,516), Fortaleza [Ceará State] (2,665) and Porto Alegre [Rio Grande do Sul State] (1,948), totaling 8,129 participants was used. These cities were focused because address information is available for their ERICA participants, which we obtained via an informed consent form for the adolescents who provided blood samples and agreed for them to be stored for future analysis. Of this total, 5,745 participants were excluded, because they were not georeferenced. This exclusion was necessary due to the low quality of the address data provided.

Authors describe the three stages of georeferencing: addresses standardization, georeferencing via the Google Maps Application Programming Interface (API), and manual intervention ^16^. The final study population included 2,384 adolescents.

### Data sources

The questionnaire completed by the adolescents to obtain ERICA participant data regarding socio-demographic characteristics and alcohol consumption was used. Data regarding urban features surrounding the household was obtained from the 2010 *Demographic Census* ^17^.

### Outcome variable

The outcome variable was alcoholic beverage consumption measured by the following question: “On how many of the past 30 days (one month) did you drink at least one glass or shot of an alcoholic beverage?”. Based on this question, a binary variable was created to identify and organize the adolescents in the sample who consumed (“1 or 2 days”, “3 to 5 days”, “6 to 9 days”, “10 to 19 days”, “20 to 29 days” and “on all 30 days”) and those who had not consumed alcohol over the previous month (“none” and “I have never drunk alcohol”). Consumption was thus characterized as having consumed an alcoholic beverage at least once in the previous 30 days, at any rate or quantity ^18^.

The question was taken from the adolescent questionnaire used in ERICA, a self-administered tool consisting of questions on socioeconomic status, employment status, smoking, alcohol consumption, physical activity assessment, medical and health history, sleep hours, eating behavior, oral health, common mental disorders, and reproductive health ^13^.

### Exposure variable

Urban physical disorder was measured according to the urban features in the 2010 *Demographic Census*, considered as exposure variables for this study, which were: number of households with street lighting, paving, street trees, manholes, accumulated rubbish on public streets, open sewers, curb, paved roads, and wheelchair ramps ^17^ ^,^ ^19^.

Based on these contextual exposure variables, measured by the census enumerator at census tract level, indicators for the surroundings were created. Firstly, circular buffers were defined, using a 100 to 250 meters radius, with the adolescents’ residences in each capital as the central point. The radius size was based on the literature ^20^. 

Values were obtained for each indicator as a weighted average of the proportion of households with a specific characteristic, in which weights were proportional to the intersection of each census tract with the buffer, since a buffer may intercept with more than one census tract. Following observation of the distribution of indicators, significant asymmetric distribution was found. Three new indicators were therefore created, based on the 75th, 80th and 90th percentile. Wheelchair ramps, accumulated rubbish on public streets and open sewer indicators were also categorized as values greater than zero, indicating the presence of these characteristics in the surroundings.

### Covariables

The adolescent sociodemographic variables were: sex (male and female); age group in years (12 to 14 and 15 to 17); race (white, mixed-race, black and other); type of school (public and private); paid work (yes and no); family structure (lives with both parents, lives with only one parent and does not live with parents); maternal and paternal education (illiterate or < 1 year, incomplete primary, complete primary or incomplete secondary, complete secondary or incomplete tertiary, and complete tertiary education); and economic class, according to criteria from the Brazilian Association of Research Companies (ABEP, acronym in Portuguese) ^21^, categorized into higher (A1, A2, B1, B2) and lower strata (C1, C2, D and E).

To adjust the regression models, the variables sex, age, family structure and the weighted average of per capita income in household surroundings were used as a proxy for individual socioeconomic status. The minimal sufficient adjustment set to estimate the total effect was obtained using the DAGitty, version 3.0 (http://www.dagitty.net/).

### Descriptive analysis

Absolute and relative frequencies were estimated to describe the population, according to sociodemographic and alcohol consumption variables, and the prevalence of alcoholic beverage consumption in the last 30 days, as well as their respective 95%CI.

### Estimating the effects

Given the study hypothesis that adolescents who reside in areas with more severe urban physical disorder are more likely to consume alcohol, a causal diagram (DAG) ^22^ of the potential relationships between exposure and outcome in the study population was created.

Total effect of the indicators of exposure to urban physical disorder on the consumption of alcohol among adolescents was estimated using Poisson regression models with robust variance. Crude and adjusted prevalence ratios (PR) were calculated with 95%CI for each exposure, controlling for possible confounding variables. When analyzing data from cross-sectional studies, Poisson models with robust variance provide good alternatives for obtaining prevalence ratio estimates adjusted for confounding variables ^23^.

To define exposure, a 100-250 meters radius around households was considered. For each exposure two models were built: the first without using the covariables (crude association); and the second adjusting for sex, age, family structure and the average per capita income in the buffer. 

For sensitivity, analyses were performed considering exposure to urban physical disorder at different cutoff points (greater than 0, greater than 75%, greater than 80% and greater than 90%), with 100-250 meters buffers. The main purpose of the choice of cutoff points was to measure the gradient of effects.

Analyses without adjusting for per capita income were also conducted to diagnose over-adjustment, since this variable is understood as a strong predictor of urban disorder. The effect of participant socioeconomic status was measured using the weighted average of per capita income in the census tracts as a proxy for individual socioeconomic status.

Statistical analyses taking the sample design described in the Vasconcellos et al. ^14^ study were perdomed, using the *survey* package of the R statistical program, version 4.1.2 (http://www.r-project.org), for the complex sample analysis.

### Ethical aspects

The study was based on ERICA data from Brazilian National Institute of Geography and Statistics (IBGE), respecting the principles contained in *Resolution n. 466/2012*, which approved the guidelines and regulatory norms for research involving humans. ERICA was conducted according to the ethical principles contained in the *Helsinki Declaration* and approved by the Ethics Research Committee of the Institute of Collective Health Studies at the Federal University of Rio de Janeiro (UFRJ; opinion n. 01/2009, process n. 45/2008) and by the Research Ethics Committee of each Federal Unit [Research Ethics Committee of the Porto Alegre Clinical Hospital, Federal University of Rio Grande do Sul (CAAE n. 45572715.8.2003.5327, opinion n. 1.230.984); Research Ethics Committee of the Institute of Studies in Public Health, Federal University of Rio de Janeiro (CAAE n. 45572715.8.1001.5286, opinion n. 1.132.879); and Research Ethics Committee of the Walter Cantídio University Hospital, Federal University of Ceará (CAAE n. 45572715.8.2001.5045, opinion n. 1.237.709). The authors filled a proposal submission form, which was then sent to the ERICA Coordination Team for authorization. 

The 2010 *Demographic Census* follows the normative principles provided for in *Law n. 5,534* of November 14, 1968. According to this law, the information is confidential and mandatory, exclusively intended for statistical purposes, and publicly accessible ^17^.

## Results

### Descriptive analysis

The sample was composed of 2,384 students, 49.6% female and 50.4% male, distributed in the age groups of 12 to 14 (52.3%) and 15 to 17 years old (47.7%). Most participants study at public schools (66.2%). Some participants (16.8%) engaged in paid work and 55.1% lived with both parents. Table 1 provides information on sample distribution and the estimated population after expanding the sample, with a description of the sociodemographic profile of the study participants.

Of this total sample, 572 participants reported drinking at least one glass (or shot) of alcohol in the previous 30 days, resulting in a general consumption prevalence of 19.6% (95%CI: 17.1; 22.1), with a higher prevalence of consumption among females (PR = 20.4, 95%CI: 17.7; 23.1). Adolescents aged 15 to 17 years had a higher prevalence of consumption (PR = 26.6, 95%CI: 22.9; 30.3) than those aged 12 to 14 (PR = 13.3, 95%CI: 10.4; 16.2).

**Table 1:** Sample sociodemographic profile and population. *Study of Cardiovascular Risks in Adolescents* (ERICA), Rio de Janeiro, Porto Alegre (Rio Grande do Sul State) and Fortaleza (Ceará State), Brazil, 2013 and 2014.

Characteristics	Sample (n = 2,384)	Estimated population (n = 875,947)	Prevalence [% (95%CI)]
Sex			
Female	1,447	434,672	49.6 (49.6; 49.6)
Male	937	441,275	50.4 (50.4; 50.4)
Age group (years)			
12 to14	937	458,257	52.3 (52.3; 52.3)
15 to 17	1,447	417,690	47.7 (47.7; 47.7)
Race			
White	1,067	369,767	42.2 (37.3; 47.1)
Mixed-race	1,028	380,986	43.5 (40.6; 46.4)
Black	184	77,874	8.9 (6.9; 10.9)
Other *	66	27,783	3.2 (2.2; 4.1)
Do not know or prefer not to answer	39	19,536	2.2 (0.9; 3.6)
Type of school			
Public	1,701	580,302	66.2 (53.3; 79.2)
Private	683	295,646	33.8 (20.8; 46.7)
Paid work			
Yes	434	147,434	16.8 (14.6; 19.1)
No	1,950	728,513	83.2 (80.9; 85.4)
Family structure			
Lives with both parents	1,326	482,858	55.1 (52.7; 57.5)
Lives with only one parent	935	349,503	39.9 (37.6; 42.2)
Does not live with parents	123	43,586	5.0 (3.6; 6.3)
Maternal education			
Illiterate or < 1 year	23	8,743	1.0 (0.4; 1.6)
Incomplete primary	336	104,548	11.9 (9.7; 14.2)
Complete primary or incomplete secondary	360	123,335	14.1 (11.7; 16.4)
Complete secondary or incomplete tertiary	678	227,721	26.0 (22.7; 29.3)
Complete tertiary	522	206,313	23.6 (16.1; 31.0)
Do not know/remember	465	205,287	23.4 (20.0; 26.9)
Paternal education **			
Illiterate or < 1 year	18	4,315	0.5 (0.2; 0.8)
Incomplete primary	116	40,545	4.6 (3.4; 5.8)
Complete primary or incomplete secondary	112	38,797	4.4 (3.6; 5.3)
Complete secondary or incomplete tertiary	228	68,387	7.8 (6.4; 9.2)
Complete tertiary	226	99,377	11.3 (7.2; 15.5)
Do not know/remember	198	84,656	9.7 (7.4; 11.9)
Economic class *** ^#^			
Higher strata	1,155	401,488	45.8 (39.9; 51.8)
Lower strata	560	191,747	21.9 (18.1; 25.6)

95%CI: 95% confidence interval.

Source: prepared by authors.

* Other categories of race: Asian and Indigenous;

** Missing data for paternal education: 61.6%;

*** Economic class according to Brazilian Association of Research Companies ^21^;

^#^ Missing data for economic class: 32.3%.

We observed a higher prevalence of consumption among adolescents who engaged in paid work (PR = 29.1, 95%CI: 23.8; 34.4) and who were more economically advantaged (PR = 22.2, 95%CI: 18.0; 26.4). There was also a higher prevalence of alcohol consumption among adolescents who did not live with their parents (PR = 24.8, 95%CI: 15.0; 34.6) and whose mothers had completed higher education (PR = 22.3, 95%CI: 13.9; 30.6). This and other information about alcohol consumption can be found in Table 2.

**Table 2:** Prevalence and confidence intervals for the consumption of alcohol by the subgroups of interest. *Study of Cardiovascular Risks in Adolescents* (ERICA), Rio de Janeiro, Porto Alegre (Rio Grande do Sul State) and Fortaleza (Ceará State), Brazil, 2013 and 2014.

Characteristics	Sample (N = 2,384)	Estimated population (n = 875,947)	Prevalence [% (95%CI)]
Total	572	168,376	19.6 (17.1; 22.1)
Sex			
Female	364	86,398	20.4 (17.7; 23.1)
Male	208	81,978	18.9 (15.5; 22.2)
Age group (years)			
12 to14	146	59,681	13.3 (10.4; 16.2)
15 to 17	426	108,695	26.6 (22.9; 30.3)
Race			
White	298	72,487	19.9 (16.8; 23.1)
Mixed-race	213	75,978	20.2 (16.7; 23.8)
Black	43	13,854	18.9 (12.5; 25.3)
Other *	10	2,468	9.0 (2.6; 15.3)
Do not know or prefer not to answer	8	3,589	19.6 (7.3; 31.8)
Type of school			
Public	353	111,267	19.6 (16.6; 22.6)
Private	219	57,109	19.7 (13.1; 26.2)
Paid work			
Yes	137	41,881	29.1 (23.8; 34.4)
No	435	126,495	17.7 (15.2; 20.2)
Family structure			
Lives with both parents	286	82,284	17.4 (14.1; 20.7)
Lives with only one parent	255	76,022	22.1 (18.3; 25.9)
Does not live with parents	31	10,070	24.8 (15.0; 34.6)
Maternal education			
Illiterate or < 1 year	5	1,588	18.2 (-2.8; 39.1)
Incomplete primary	64	19,210	18.7 (14.0; 23.4)
Complete primary or incomplete secondary	79	20,059	16.5 (12.5; 20.6)
Complete secondary or incomplete tertiary	169	45,155	20.1 (15.0; 25.1)
Complete tertiary	162	45,471	22.3 (13.9; 30.6)
Do not know/remember	93	36,893	18.8 (13.5; 24.2)
Paternal education **			
Illiterate or < 1 year	1	83	2.0 (-1.8; 5.8)
Incomplete primary	22	6,874	17.0 (9.5; 24.6)
Complete primary or incomplete secondary	21	7,263	19.0 (9.6; 28.4)
Complete secondary or incomplete tertiary	58	14,356	21.4 (15.1; 27.6)
Complete tertiary	68	17,564	17.8 (8.7; 26.9)
Do not know/remember	27	11,946	14.6 (9.0; 20.2)
Economic class *** ^#^			
Higher strata	333	88,461	22.2 (18.0; 26.4)
Lower strata	117	34,712	18.5 (14.6; 22.4)

95%CI: 95% confidence interval.

Source: prepared by authors.

* Other categories of race: Asian and Indigenous;

** Missing data for paternal education: 61.6%;

*** Economic class according to Brazilian Association of Research Companies ^21^;

^#^ Missing data for economic class: 32.3%.

### Effect estimates

Table 3 presents the results of the crude and adjusted analyses for sex, age, average per capita income in household surroundings and family structure. The associations varied according to the household surroundings indicator and buffer size. 

**Table 3:** Association between household surroundings indicators, with 100 and 250 meters buffers, and alcohol consumption by adolescents. *Study of Cardiovascular Risks in Adolescents* (ERICA), Rio de Janeiro, Porto Alegre (Rio Grande do Sul State) and Fortaleza (Ceará State), Brazil, 2013 and 2014.

Indicators *	Crude estimates		Adjusted estimates **	
	Buffer 100m	Buffer 250m	Buffer 100m	Buffer 250m
	PR (95%CI)	PR (95%CI)	PR (95%CI)	PR (95%CI)
Lighting				
p75	1.00 (0.81; 1.23)	1.18 (0.97; 1.43)	0.88 (0.74; 1.06)	1.04 (0.86; 1.26)
p80	1.04 (0.82; 1.31)	1.11 (0.87; 1.40)	0.93 (0.75; 1.15)	0.95 (0.77; 1.17)
p90	1.24 (0.98; 1.57)	1.17 (0.87; 1.59)	1.15 (0.91; 1.45)	1.04 (0.81; 1.34)
Paving				
p75	1.15 (0.93; 1.42)	1.25 (1.00; 1.57)	1.04 (0.86; 1.26)	1.09 (0.88; 1.34)
p80	1.18 (0.93; 1.48)	1.21 (0.93; 1.58)	1.07 (0.86; 1.33)	1.02 (0.80; 1.31)
p90	1.19 (0.91; 1.55)	1.28 (0.96; 1.70)	1.05 (0.81; 1.36)	1.09 (0.83; 1.41)
Paved roads				
p75	1.15 (0.93; 1.42)	1.39 (1.15; 1.67)	1.00 (0.83; 1.21)	1.24 (1.02; 1.50)
p80	1.14 (0.90; 1.44)	1.41 (1.16; 1.71)	1.00 (0.81; 1.23)	1.23 (1.02; 1.49)
p90	1.26 (0.95; 1.68)	1.40 (1.06; 1.85)	1.09 (0.83; 1.43)	1.15 (0.92; 1.44)
Curbs				
p75	1.15 (0.93; 1.43)	1.30 (1.09; 1.57)	0.99 (0.82; 1.19)	1.15 (0.97; 1.35)
p80	1.19 (0.94; 1.49)	1.32 (1.09; 1.61)	1.05 (0.85; 1.30)	1.16 (0.99; 1.36)
p90	1.22 (0.94; 1.60)	1.28 (0.94; 1.75)	1.09 (0.84; 1.41)	1.07 (0.85; 1.35)
Manholes				
p75	1.43 (1.10; 1.86)	1.31 (1.02; 1.70)	1.27 (0.92; 1.76)	1.19 (0.89; 1.60)
p80	1.41 (1.05; 1.89)	1.37 (1.04; 1.81)	1.24 (0.85; 1.79)	1.19 (0.86; 1.65)
p90	1.36 (0.96; 1.93)	1.58 (1.21; 2.08)	1.19 (0.79; 1.79)	1.36 (1.01; 1.84)
Street trees				
p75	1.16 (0.85; 1.57)	1.20 (0.91; 1.58)	1.00 (0.78; 1.30)	1.00 (0.77; 1.29)
p80	1.15 (0.83; 1.60)	1.20 (0.87; 1.65)	0.99 (0.75; 1.32)	0.99 (0.74; 1.33)
p90	1.00 (0.63; 1.58)	1.15 (0.77; 1.73)	0.81 (0.56; 1.18)	0.93 (0.64; 1.35)
Ramps				
p75	0.91 (0.72; 1.16)	1.19 (0.88; 1.62)	0.79 (0.62; 0.99)	0.91 (0.75; 1.10)
p80	1.08 (0.77; 1.53)	1.30 (0.95; 1.76)	0.89 (0.69; 1.14)	0.99 (0.82; 1.21)
p90	1.16 (0.79; 1.69)	1.28 (0.91; 1.81)	0.87 (0.65; 1.16)	0.85 (0.68; 1.07)
Greater than 0	1.29 (0.98; 1.70)	1.12 (0.85; 1.48)	1.15 (0.91; 1.45)	1.00 (0.80; 1.25)
Sewage				
p75	0.87 (0.64; 1.17)	0.89 (0.67; 1.18)	0.91 (0.67; 1.24)	0.94 (0.70; 1.25)
p80	0.98 (0.71; 1.34)	0.77 (0.54; 1.10)	1.04 (0.75; 1.43)	0.82 (0.57; 1.03)
p90	0.90 (0.54; 1.50)	0.99 (0.64; 1.53)	0.99 (0.58; 1.68)	1.03 (0.63; 1.68)
Greater than 0	0.76 (0.60; 0.97)	0.69 (0.55; 0.86)	0.84 (0.68; 1.02)	0.80 (0.66; 0.97)
Rubbish				
p75	0.99 (0.77; 1.26)	0.87 (0.72; 1.04)	1.04 (0.82; 1.31)	0.94 (0.79; 1.11)
p80	0.80 (0.64; 0.99)	0.98 (0.79; 1.20)	0.85 (0.70; 1.03)	1.06 (0.88; 1.29)
p90	0.77 (0.56; 1.06)	0.89 (0.68; 1.16)	0.82 (0.61; 1.11)	0.97 (0.74; 1.27)
Greater than 0	0.86 (0.67; 1.10)	0.79 (0.64; 0.98)	0.91 (0.72; 1.15)	0.87 (0.72; 1.05)

95%CI: 95% confidence interval; p75: 75th percentile; p80: 80th percentile; p90: 90th percentile; PR: prevalence ratio.

Source: prepared by authors.

Note: statistically significant values are shown in bold.

* Exposures are binary and values are cut-off points: p75 = greater than 75%; p80 = greater than 80%; p90 = greater than 90%; greater than 0 = values greater than 0;

** Estimates adjusted for: sex, age group, family structure and per capita income in the household surroundings.

Adolescents who live in environments with paved roads and manholes were more likely to consume alcohol, with adjusted PR = 1.24 (95%CI: 1.02; 1.50) and adjusted PR = 1.36 (95%CI: 1.01; 1.84), respectively, statistically significant in the 250-meter buffer.

Conversely, adolescents living in areas with wheelchair ramps and open sewage were less likely to consume alcohol, with an adjusted PR = 0.79 (95%CI: 0.62; 0.99) and adjusted PR = 0.80 (95%CI: 0.66; 0.97), respectively, with the association with wheelchair ramps being statistically significant in the 100-meter buffer and the association with open sewage statistically significant in the 250-meter buffer.

Moreover, when we examined the adjusted values, results suggested a positive association between adolescents who live in regions with street lighting, paved roads and kerbs, and alcohol consumption, while living in locations with street trees and accumulated rubbish on public streets was inversely associated with alcohol consumption. 

## Discussion

### Prevalence of alcohol consumption among adolescents in Brazilian capitals

The results of the prevalence of alcohol consumption among adolescents in the three studied Brazilian capital cities are similar to those found in the literature. The consumption of alcoholic beverages in the last 30 days was higher among girls and adolescents aged 15 to 17 years. The same findings can be observed in a study carried out with data from 2019 PeNSE, which observed a higher prevalence of alcohol consumption among girls (30.1%, 95%CI: 29.2; 31.0) and in older adolescents (30.1%, 95%CI: 29.2; 31.0) ^8^.

Although examples of studies in Brazil ^11^ and around the world ^24^ ^,^ ^25^ show higher prevalence of alcohol consumption among males, our finding is supported by the rapid reduction in the disparity in substance use between genders, with girls equaling or surpassing boys in smoking rates, alcohol consumption, and electronic cigarette use at age 15, according to data from the World Health Organization (WHO) ^26^.

The higher prevalence of alcohol consumption in older adolescents can be explained by their proximity to adulthood, which is characterized by the search for autonomy and personal identity, the search for new experiences and sensations, in addition to peer influence ^27^.

We observed a higher prevalence of consumption among adolescents who worked, were more economically advantaged, and whose mothers had completed tertiary education. These are important socioeconomic markers (including education ^28^) that may suggest adolescents’ access to a higher income. Better income conditions can provide greater access to the consumption of alcoholic beverages, although there is no consensus in the literature regarding family income and alcoholic beverage consumption by adolescents, as some studies report greater consumption in families with a higher income, while other studies related alcohol consumption with lower incomes ^11^.

Adolescents who did not live with both parents were also more likely to consume alcohol.

A study carried out in Brazil ^29^ observed that adolescents who had parents who showed negligent parental behavior were more likely to resort to polydrug consumption. This study ^29^, which was also developed with data from PeNSE 2019, highlighted the importance of parental monitoring in preventing alcohol consumption and other drugs by adolescents. Parents can avoid adolescent’s consumption of alcohol by paying attention to their children, seeking information about their free time, their activities, their friends, and investing in dialogue and open communication ^29^.

### Urban physical disorder and alcohol consumption among adolescents in Brazilian capitals

In this study, we investigated the total effect of urban physical disorder indicators and alcohol consumption among adolescents in three Brazilian capitals. We observed a higher probability of alcohol consumption among adolescents who lived in areas with paved roads and manholes. Those who lived in environments with wheelchair ramps and, surprisingly, areas with open sewages proved to be less likely to consume alcohol.

The positive association between adolescents who lived in places with a higher proportion of paved roads and manholes and alcohol consumption indicates the possibility that these areas have an infrastructure that enables mobility and interaction between people and the environment ^30^. Furthermore, they may have a structure that encourages a greater density of commercial establishments, resulting in an increase in the availability of alcoholic beverages ^31^ ^,^ ^32^.

A study by Carvalho et al. ^33^ demonstrated a significant association between the current alcohol consumption and the density of establishments located within a 200-meter radius from the teenagers’ residences , requiring public policy actions linked to the inspection and control operation of establishments that sell alcoholic beverages.

The inverse association between wheelchair ramps and alcohol consumption among adolescents may suggest that a greater proportion of this indicator reflects better urban planning and infrastructure conditions, since there are still many regions in Brazil that do not have adequate and efficient accessibility structures ^34^. In this way, the presence of ramps characterize healthier and health-friendly environments that promote a better quality of life for its residents ^35^.

Surprisingly, adolescents who live in environments with open sewages on public streets were found to consume less alcohol. According to Aguiar et al. ^36^, the higher the family income, the better the housing conditions and the more appropriate the access to various essential services, including basic sanitation, adequate water supply, sewage disposal and regular waste collection.

For Massa et al. ^37^, a lack of sewage disposal is more common in regions occupied by populations with low incomes and low levels of education, which suggests that people from a lower socioeconomic level have less purchasing power and therefore find it more difficult to obtain alcohol to consume, as a higher prevalence of alcohol consumption has been related to better socioeconomic conditions ^38^, which we also observed in our study in which a higher prevalence of alcohol consumption was observed among adolescents who worked.

Another issue is that a lack of basic sanitation may be a feature of housing in more violent areas, which may relate to poverty indicators and changes to the population’s habits ^39^. In Brazil, the expansion of the city peripheries and the socio-spatial segregation related to an absence of, or precarious, city infrastructure has caused significant damage to economic dynamics and to people’s quality of life regarding mobility and urban accessibility ^40^. 

### Strengths and limitations

Our results suggest the importance of research into the relationship between urban characteristics and health. However, this study has certain limitations. The sample was only composed of adolescents attending school; we did not include adolescents who were not enrolled in school, who may be more prone to alcohol consumption, since some studies suggest that the consumption of high levels of alcohol in adolescents may lead to significant school losses, such as truancy and school dropout ^41^. However, the results of research undertaken in school can provide tools for health promotion and preventive public policies aimed at this target audience ^42^.

Caution is needed when generalizing these findings, given that the individual data was collected in 2013 and 2014, and only refers to three Brazilian capitals, while the sample was only composed of adolescents who participated in blood collection, allowed us to identify their residential addresses, and studied in the morning, because they needed to fast prior to the tests. Furthermore, factors such as residential mobility and urban segregation may also influence the various types of exposure to urban disorder. Overall, the more restricted the specific environment, the greater the exposure to the (positive and negative) characteristics in the surroundings ^43^.

As this study is based on responses to a self-reported questionnaire ^13^, some questions may not have been adequately understood or participants may have omitted risky behaviors when filling them out, which may result in the underestimation of alcohol consumption in the sample, in addition to the possibility of generating inconsistencies in the results. However, well-structured self-reported questionnaires are a robust tool for collecting data, increasing the credibility of the results ^44^. Similar questionnaires have been used in other countries ^24^.

The exposure data was based on the 2010 census which, although it was the most up to date at the time of the study, may limit representation of the real situation, due to the lag in time. Furthermore, the use of circular buffers to define exposure may include uninhabited areas, which the adolescent does not effectively move through ^33^. Moreover, georeferencing performs poorer in less developed urban areas, which may have hampered our georeferencing of participant addresses. However, although the sample was significantly reduced after georeferencing, with methodological care in expanding the sample it was possible to find results consistent with findings estimated in the entire study population ^18^.

Using characteristics of household surroundings enables an analysis of positive or negative responses regarding the existence of such items around residences, however, it does not provide a qualitative view of these characteristics. Thus, it is not possible to assess the quality of the maintenance or conservation of infrastructure, which might have produced different results. However, the use of exposure data, collected systematically by a census enumerator, provided important contributions to the study. Studies that explore the use of qualitative methods can contribute to a better understanding of the observed associations.

## Conclusions

Bearing in mind the challenges posed by a study that seeks to investigate the association between urban physical disorder and a health outcome, due to the scarce methodological references to measure urban disorder, this study provides an innovative approach to measuring urban physical disorder and its relation to alcohol consumption in adolescents. 

Around the world, urban physical disorder is the product of intense and rapid urbanization, and it is important to understand its influence on health, particularly in developing countries such as Brazil; activities aimed at caring for the Brazilian urban population have become urgent. This study contemplated several important aspects and added epidemiological findings about the association between urban physical disorder and the consumption of alcohol among adolescents. 

The prevalence of alcohol consumption among adolescents demands attention from the public health sector and investment in studies aimed at accumulating knowledge. Examining individual and contextual factors, and their potential relationships with consumption, may be an important strategy for a broader understanding of the current health situation of adolescents who live in urban environments. 

Given the lack of scientific evidence on this subject in Brazil, these results could support an understanding of the association between aspects of urban physical disorder and the consumption of alcohol, generating information to support interventions aimed at reducing adolescent use of alcohol, and encouraging interest in the subject for the development of new studies that will examine the topic in depth and extend it to other populations.
